# Semantic and Geometric-Aware Day-to-Night Image Translation Network

**DOI:** 10.3390/s24041339

**Published:** 2024-02-19

**Authors:** Geonkyu Bang, Jinho Lee, Yuki Endo, Toshiaki Nishimori, Kenta Nakao, Shunsuke Kamijo

**Affiliations:** 1 Emerging Design and Informatics Course, Graduate School of Interdisciplinary Information Studies, The University of Tokyo, 4 Chome-6-1 Komaba, Meguro-ku, Tokyo 153-0041, Japan; leejinho@kmj.iis.u-tokyo.ac.jp; 2 Department of Information and Communication Engineering, Graduate School of Information Science and Technology, The University of Tokyo, 7 Chome-3-1 Hongo, Bunkyo-ku, Tokyo 113-8656, Japan; endou@kmj.iis.u-tokyo.ac.jp; 3 Mitsubishi Heavy Industries Machinery Systems, Ltd., 1 Chome-1-1 Wadasaki-cho, Hyogo-ku, Kobe 652-8585, Japan; toshiaki.nishimori.23@mhi.com; 4 Mitsubishi Heavy Industries, Ltd., 1 Chome-1-1 Wadasaki-cho, Hyogo-ku, Kobe 652-8585, Japan; kenta.nakao.r2@mhi.com; 5 Institute of Industrial Science, The University of Tokyo, 4 Chome-6-1 Komaba, Meguro-ku, Tokyo 153-0041, Japan

**Keywords:** image-to-image translation, domain adaptation, data augmentation, generative adversarial networks, generative model, deep learning

## Abstract

Autonomous driving systems heavily depend on perception tasks for optimal performance. However, the prevailing datasets are primarily focused on scenarios with clear visibility (i.e., sunny and daytime). This concentration poses challenges in training deep-learning-based perception models for environments with adverse conditions (e.g., rainy and nighttime). In this paper, we propose an unsupervised network designed for the translation of images from day-to-night to solve the ill-posed problem of learning the mapping between domains with unpaired data. The proposed method involves extracting both semantic and geometric information from input images in the form of attention maps. We assume that the multi-task network can extract semantic and geometric information during the estimation of semantic segmentation and depth maps, respectively. The image-to-image translation network integrates the two distinct types of extracted information, employing them as spatial attention maps. We compare our method with related works both qualitatively and quantitatively. The proposed method shows both qualitative and qualitative improvements in visual presentation over related work.

## 1. Introduction

Autonomous driving systems require effective and secure operation under various visibility conditions. The functionality of these systems is heavily dependent on their perception tasks, which have seen significant improvements in accuracy through advances in deep learning in recent years. Despite these advances, challenges persist in addressing perception tasks under poor visibility conditions (e.g., nighttime, rain, and fog). The primary obstacle stems from an imbalance in the amount of available data for each scenario. Deep-learning-based models, reliant on substantial datasets and annotations for training, often encounter difficulties due to the scarcity of relevant data for adverse visibility situations. Although numerous datasets have been created, most are concentrated on clear daytime conditions, making it impractical to collect and annotate data for every conceivable traffic scene and visibility scenario.

To address this challenge, researchers [1,2,3,4,5,6,7] have increasingly utilized synthetic data (e.g., computer graphic images from sources such as video games and simulators) to diversify the datasets. Despite the advantages of easy dataset creation for various scenarios, there remains a disparity between synthetic and real-world data. Consequently, deep-learning-based models (e.g., depth estimation, semantic segmentation, and camera pose estimation) trained with synthetic data may exhibit decreased performance in real-world applications. Efforts to enhance the photorealism [8,9,10,11] have been made, but the usability of models trained on synthetic data for autonomous driving systems remains a challenge.

In contrast, day-to-night image translation offers a solution by creating realistic nighttime data while preserving the objects, structure, and perspective. This process involves translating annotated daytime images into nighttime images, enabling the utilization of daytime image labels for the translated nighttime images. This facilitates the creation of nighttime datasets.

Numerous contemporary image translation methods leverage generative adversarial networks (GANs) [12], a robust framework for the training of generative models. However, it is challenging to obtain paired data for model training in traffic scenes (i.e., daytime and nighttime image pairs where every corresponding point is the same, except for the time of day). Consequently, this paper adopts unsupervised image-to-image translation methods to address the lack of paired data.

**Figure 1:**
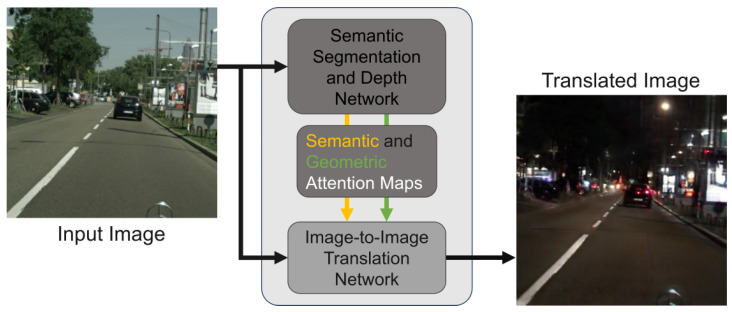
The concept of our proposed method. Semantic and geometric information of input images is extracted as feature maps by pre-trained semantic segmentation and depth network. Utilizing attention modules, semantic and geometric spatial attention maps are deduced from these feature maps. Subsequently, both semantic and geometric attention maps are integrated into the image-to-image translation network.

**Figure 2:**
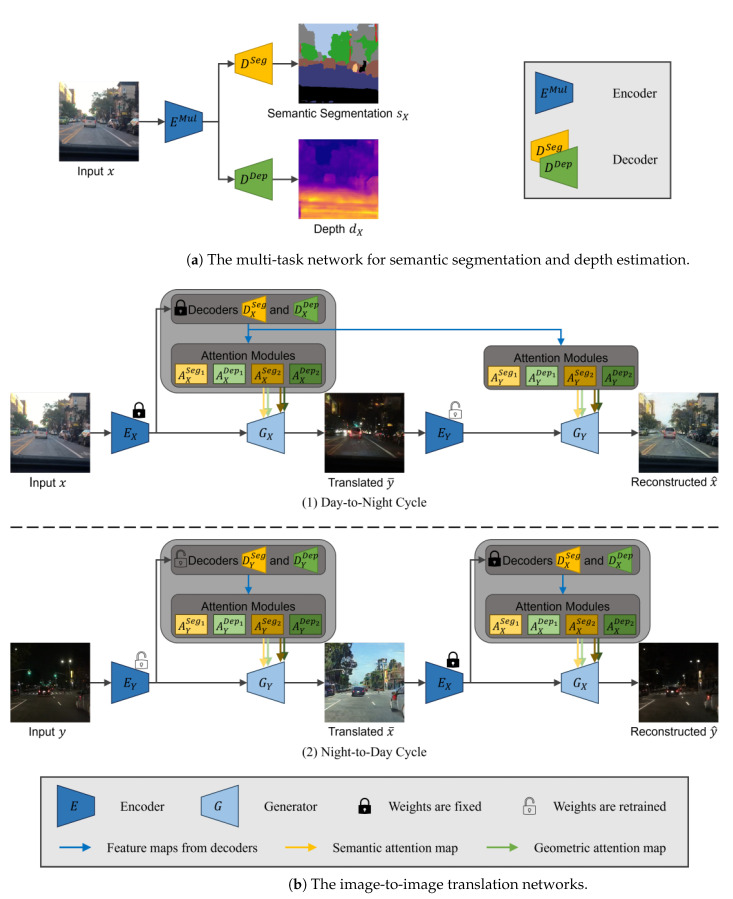
The overview of the proposed method. The training process consists of two distinctive steps, (**a**) the semantic segmentation and depth multi-task network and (**b**) the image-to-image translation network. During the second step, encoders and decoders utilize the pre-trained parameters obtained in the first step, and attention modules infer spatial attention maps from the feature maps of decoders. Throughout only the day-to-night cycle, the feature maps of decoders are shared between the image translation and reconstruction processes.

In this paper, we introduce an unsupervised day-to-night image translation model based on GANs [12] as a data augmentation technique. The translation of daytime images to nighttime poses a formidable challenge. This requires not only accurate color adjustment but also the consideration of semantic and geometric information at the pixel level. The goal is to achieve consistent transformation in semantic and geometric information while allowing for diverse style conversion. As shown in Figure 1, our model sets a hypothesis. We assume that the semantic and geometric information can be extracted from semantic segmentation and depth estimation. We first train the multi-task network, which estimates the semantic segmentation and depth (Figure 2a). The trained parameters of the multi-task network are utilized for the encoder and decoders of the image translation networks (Figure 2b). The attention module infers the attention map using the feature map extracted from the decoder as input. Leveraging the semantic segmentation and depth multi-task estimation network’s capacity to extract vital semantic and geometric information, the attention modules are able to infer semantic and geometric attention maps along the spatial dimension.

Our contributions can be summarized as follows.

- We propose a semantic- and geometric-aware image-to-image translation network that adopts semantic segmentation and depth estimation guided attention modules. To the best of our knowledge, this is the first work that utilizes both semantic segmentation and depth information in image-to-image translation.
- We introduce the semantic segmentation and depth estimation guided attention modules and adopt them for image-to-image translation. Our method does not require annotations for the target domain.
- The proposed method generates better results both quantitatively and qualitatively in our experiments; it outperforms the related work in two distinct evaluation metrics.

Our method is trained with two different authentic datasets (i.e., Berkeley Deep Drive [13], Cityscapes [14]) at the same time.

## 2. Related Work

### 2.1. Generative Adversarial Networks (GANs)

Generative models within the realm of deep learning, particularly those based on the framework of generative adversarial networks (GANs) [12], have received significant attention. The fundamental structure of GANs [12] involves two adversarial networks (i.e., a generator and a discriminator) engaged in a competitive training process. The generator aims to produce data that the discriminator perceives as real, leading to a continuous interplay between the two networks. Subsequent to the introduction of GANs [12], various enhancements and alternative versions have been proposed to further improve their capabilities. cGAN [15] is one such advancement, introducing a conditional approach by incorporating additional input layers to condition the data. This allows the explicit generation of outputs based on specified conditions. Combining GANs [12] with auto-encoders [16], VAE/GAN [17] and VEEGAN [18] represent innovative approaches. These models leverage the strengths of both GANs and auto-encoders, with the aim of enhancing the overall generative process. In pursuit of better training objectives, alternative loss functions have been explored. LSGA [19] addresses the vanishing gradients problem by utilizing the least-squares loss function for the discriminator. This adjustment helps to stabilize the training process and improve the overall performance of the GAN [12] model. WGAN [20] introduces a different training objective by adopting the Wasserstein distance between the distributions of generated and real data. This alternative approach aims to overcome the limitations associated with traditional GANs’ training objectives.

### 2.2. Image-to-Image Translation Network

Pix2Pix [21] made significant strides as the initial unified framework for paired image-to-image translation, using cGAN [15]. In more recent developments, there has been a shift towards unsupervised image-to-image translation methods, which operate without a reliance on paired data. To tackle the inherent challenges of this ill-posed problem, various approaches have adopted the cycle consistency constraint. This constraint ensures that the translated data can be accurately reconstructed back to the source domain [22,23,24,25]. Some methods assume a shared latent space among images in different domains. CoGAN [26] features two generators with shared weights, producing images from different domains using the same random noise. UNIT [27], building upon CoGAN and incorporating VAE/GAN [17], maps each domain into a common latent space. Additionally, the exploration of multimodal image-to-image translation methods has gained traction [28,29,30,31,32,33]. However, these methods tend to exhibit suboptimal results when confronted with images from domains with substantial differences, such as daytime and nighttime, as they often lose instance-level information.

### 2.3. Day-to-Night Image Translation Network

Focusing on domain adaptation between daytime and nighttime images is crucial in enhancing the performance of various perception tasks, such as object detection [34], semantic segmentation [35,36], and localization [37]. Some works have attempted to boost deep network training for specific [38] or multiple [39] tasks. Some methods utilize semantic segmentation for additional information. SG-GAN [40] adopts semantic-aware discriminators, using semantic information to distinguish generated images from real ones. SemGAN [41] and Ramirez et al. [42] take a distinctive approach by inferring semantic segmentation from the translated images, thereby enforcing semantic consistency during the translation process. This emphasis on semantic information contributes to the overall perceptual coherence of the generated images. AugGAN [43,44] is a multi-task network designed for both day-to-night image translation and semantic segmentation estimation. This integrated approach reflects a comprehensive strategy in which the generator simultaneously learns the information about image translation between day and night and semantic segmentation.

### 2.4. Attention Mechanism

Attention mechanisms weight the parameters of deep learning models based on features extracted from input images. RAM [45] was initially proposed in the field of computer vision, introducing a method to recurrently estimate spatial attention and update the network. SENet [46] and ECA Net [47] introduced channel attention networks. Subsequent research has demonstrated advancements in spatial [48,49] or channel [50,51] attention. Some studies have proposed inferring attention maps along spatial and channel dimensions [52,53,54,55,56]. More recently, self-attention [57,58,59] and Transformer [60,61,62] have been introduced into computer vision, rapidly advancing the field.

## 3. Proposed Method

In this section, we propose a semantic- and geometric-aware day-to-night image translation method based on the CycleGAN framework [22]. When translating daytime images to nighttime ones, both descriptions of light sources with semantic properties and expressions of darkness according to geometric distances are required. As shown in Figure 2, the proposed method aims to extract semantic and geometric information from input images and apply it to the image-to-image translation network with the attention mechanism.

We assume that semantic and geometric information can be acquired from semantic segmentation and depth estimation processes, respectively. We first train the semantic segmentation and depth multi-task network. After, this trained multi-task network is utilized in the image translation phase to extract semantic and geometric information as feature maps. Subsequently, semantic and geometric attention maps are generated from the feature maps of the decoders. Finally, the attention maps are applied to image-to-image translation networks.

Here, we denote the domains of the daytime and nighttime RGB images by *X* and *Y*, respectively.

### 3.1. Semantic Segmentation and Depth Estimation

Figure 2a provides an overview of the multi-task network for the estimation of semantic segmentation and depth. The multi-task network first encodes authentic daytime images x∈X into latent representations via the encoder $E^{Mul}$; then, the decoders $D^{Seg}$ and $D^{Dep}$ estimate semantic segmentation maps $s_{X}$ and depth maps $d_{X}$, respectively, from the latent representation: (1) semantic segmentation map $s_{X}=D^{Seg}\left(E^{Mul}\left(x\right)\right)$ and (2) depth map $d_{X}=D^{Dep}\left(E^{Mul}\left(x\right)\right)$.

The trained parameters of the multi-task network are utilized in the image-to-image translation network.

### 3.2. Image Translation

The overall framework of our proposed method is depicted in Figure 2b. The framework consists of two opposite cycles (i.e., day-to-night cycle and night-to-day cycle) and each of them is a coupled image-to-image translation network. For each cycle, we refer to the translation from real images as translation and the translation from translated images as reconstruction.

#### 3.2.1. Image-to-Image Translation Network

The day-to-night image translation network consists of one encoder $E_{X}$, one generator $G_{X}$, two decoders for semantic segmentation $D_{X}^{Seg}$, depth $D_{X}^{Dep}$, and four CBAM [55] modules ${\left\{A_{X}^{k}\right\}}_{k\in \{Seg_{1},Dep_{1},Seg_{2},Dep_{2}\}}$. The encoder $E_{X}$ and the decoders $D_{X}^{Seg}$ and $D_{X}^{Dep}$ utilize the parameters of the encoder $E^{Mul}$ and the decoders $D^{Seg}$ and $D^{Dep}$ of Section 3.1.

**Figure 3:**
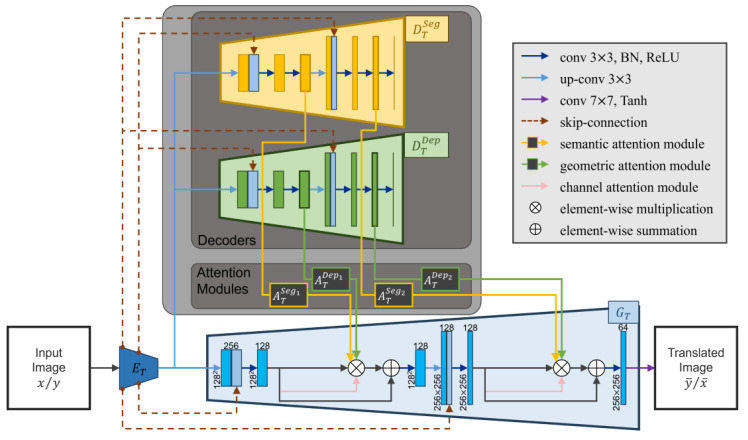
Structure of the semantic and geometric attention module. The proposed method adopts four spatial attention modules (i.e., $A_{T}^{Seg_{1}}$, $A_{T}^{Dep_{1}}$, $A_{T}^{Seg_{2}}$, and $A_{T}^{Dep_{2}}$, where *T* indicates time domain T∈{X,Y}). Each attention module infers a spatial attention map from the corresponding feature map. Here, two different sizes of feature maps are utilized from each decoder (i.e., semantic segmentation and depth decoder). The resultant attention maps are then mapped to their correspondingly sized feature maps within the generator.

As shown in Figure 3, the decoders $D_{X}^{Seg}$ and $D_{X}^{Dep}$ are utilized for the extraction of feature maps ${\left\{f_{X}^{k}\in R^{H\times W\times C}\right\}}_{k\in \{Seg_{1},Dep_{1},Seg_{2},Dep_{2}\}}$. Then, the attention maps are generated from these feature maps: ${\left\{A_{X}^{k}\left(f_{X}^{k}\right)=:m_{X}^{k}\in R^{H\times W}\right\}}_{k\in \{Seg_{1},Dep_{1},Seg_{2},Dep_{2}\}}=:M_{X}$. The inferred attention maps $M_{X}$ are applied to the generator $G_{X}$ by pixel-wise multiplication. Additionally, channel attention maps are inferred and implemented within the generator $G_{X}$.

In each cycle, authentic images {x,y} are translated into $\left\{\bar{y},\bar{x}\right\}$ by the image-to-image translation network with the semantic and geometric attention maps: $\bar{y}=G_{X}\left(E_{X}\left(x\right),M_{X}\right)$, $\bar{x}=G_{Y}\left(E_{Y}\left(y\right),M_{Y}\right)$.

The night-to-day image translation network is structured in the same way. Here, the transferred parameters of the day-to-night image translation networks $E_{X}$, $D_{X}^{Seg}$, and $D_{X}^{Dep}$ are fixed, and only those of the night-to-day image translation networks $E_{Y}$, $D_{Y}^{Seg}$, and $D_{Y}^{Dep}$ are retrained during the training of the image translation network.

Moreover, discriminators $\left\{Disc_{X},Disc_{Y}\right\}$ are defined for each domain to determine whether a daytime or nighttime image is real or fake.

#### 3.2.2. Sharing of Semantic and Geometric Feature Maps

Sharing semantic and geometric information during the image translation cycle is considered plausible, given the consistency observed in most scene elements before and after the image translation. However, challenges arise because the multi-task networks $E^{Mul}$, $D^{Seg}$, and $D^{Dep}$ are not trained on nighttime images, which can cause poor accuracy in semantic segmentation and depth estimation from nighttime images. Therefore, during the reconstruction phase, from translated images $\left\{\bar{y},\bar{x}\right\}$ to reconstructed images $\left\{\hat{x},\hat{y}\right\}$, the feature maps of semantic segmentation and depth decoders are shared only within the day-to-night cycle. In contrast, these feature maps are separately estimated in the night-to-day cycle, as shown in Figure 2b: $\hat{x}=G_{Y}\left(E_{Y}\left(\bar{y}\right),M_{X}\right)$, $\hat{y}=G_{X}\left(E_{X}\left(\bar{x}\right),{\bar{M}}_{X}\right)$, where ${\bar{M}}_{X}={\left\{A_{X}^{k}\left({\bar{f}}_{X}^{k}\right)\right\}}_{k\in \{Seg_{1},Dep_{1},Seg_{2},Dep_{2}\}}$, and ${\bar{f}}_{X}^{k}$ is the feature map extracted from translated images $\bar{x}$.

### 3.3. Training Networks

The proposed method follows a two-step training process. In the initial step, multi-task networks responsible for estimating semantic segmentation and depth maps (i.e., $\left\{E^{Mul},D^{Seg},D^{Dep}\right\}$) are trained exclusively on daytime data. In the subsequent step, the parameters of the initially trained networks $\left\{E^{Mul},D^{Seg},D^{Dep}\right\}$ are transferred to the image translation networks $\left\{E_{X},E_{Y}\right\}$, $\left\{D_{X}^{Seg},D_{Y}^{Seg}\right\}$, and $\left\{D_{X}^{Dep},D_{Y}^{Dep}\right\}$. Following this transfer, the day-to-night and night-to-day image translation networks $\left\{T_{X\to Y}:=G_{X}\left(E_{X}\left(\cdot \right)\right),T_{X\to Y}:=G_{Y}\left(E_{Y}\left(\cdot \right)\right)\right\}$ and the discriminators $\left\{Disc_{X},Disc_{Y}\right\}$ are trained using the CycleGAN framework [22]. During the training of the image translation networks, the transferred parameters for the daytime domain networks, specifically $\left\{E_{X},D_{X}^{Seg},D_{X}^{Dep}\right\}$, remain fixed. At the same time, the networks associated with the nighttime domain, denoted as $\left\{E_{Y},D_{Y}^{Seg},D_{Y}^{Dep}\right\}$, undergo a retraining process. This selective retraining allows the model to adapt and fine-tune its parameters for the unique characteristics and challenges posed by nighttime data.

#### 3.3.1. Semantic Segmentation and Depth Estimation Network

For the first step, we train the multi-task networks $\left\{E^{Mul},G^{Seg},G^{Dep}\right\}$ on the daytime images *x*. In this step, we employ multi-class cross-entropy loss $l_{mce}$ for the training of semantic segmentation and L2 loss for the training of depth estimations. Let *S* and *D* be the domains of semantic segmentation and depth labels, respectively. The mathematical expressions for the loss function of each task are given by

(1) $$L_{Seg}=E_{x∼X,s∼S}\left[l_{mce}\left(D^{Seg}\left(E^{Mul}\left(x\right)\right),s\right)\right]$$

(2) $$L_{Dep}=E_{x∼X,d∼D}\left[∥D^{Dep}\left(E^{Mul}\left(x\right)\right)-d{∥}_{2}\right]$$

The overall loss function is as follows:

(3) $$L_{Mul}=L_{Seg}+L_{Dep}$$

#### 3.3.2. Image-to-Image Translation Network

As the second step, we train the image-to-image translation networks. The image translation generators adopt the attention maps inferred from the feature maps of the semantic segmentation and depth estimation networks. Attention modules (CBAM [55]) are jointly trained with encoders and generators. Following the CycleGAN framework [22], we adopt the loss functions as follows to train the image translation network with unpaired data.

#### Adversarial Loss

Adversarial losses are implemented for both day-to-night and night-to-day image translation networks, seeking to minimize the distributional gap between translated images and targets. The objectives of the image translation networks $\left\{T_{X\to Y},T_{Y\to X}\right\}$ and their corresponding discriminators $\left\{Disc_{Y},Disc_{X}\right\}$ are expressed as follows:

(4) $$\begin{array}{cc} L_{adv1} & =E_{y∼Y}\left[\log\left(Disc_{Y}\left(y\right)\right)\right]\\ \; & \;+E_{x∼X}\left[\log\left(1-Disc_{Y}\left(T_{X\to Y}\left(x,M_{X}\right)\right)\right)\right] \end{array}$$

(5) $$\begin{array}{cc} L_{adv2} & =E_{x∼X}\left[\log\left(Disc_{X}\left(x\right)\right)\right]\\ \; & \;+E_{y∼Y}\left[\log\left(1-Disc_{X}\left(T_{Y\to X}\left(y,M_{Y}\right)\right)\right)\right] \end{array}$$

#### Cycle Consistency Loss

The cycle consistency loss is introduced to prevent the image translation network from generating arbitrary images in the target domain, regardless of the input images. The primary goal is to alter only the time of day, while preserving all other elements of the scene. To accomplish this, we apply constraints to guarantee the alignment between the input image and the reconstructed image. The cycle consistency loss is mathematically expressed as follows:

(6) $$\begin{array}{cc} L_{cyc} & =E_{x∼X}\left[{∥T_{Y\to X}\left(T_{X\to Y}\left(x,M_{X}\right),M_{X}\right)-x∥}_{1}\right]\\ \; & \;+E_{y∼Y}\left[{∥T_{X\to Y}\left(T_{Y\to X}\left(y,M_{Y}\right),{\bar{M}}_{X}\right)-y∥}_{1}\right] \end{array}$$

#### Identity Loss

The identity loss requires that the image translation networks exclusively translate the source domain images and not those from the target domain. The objective is expressed as follows:

(7) $$\begin{array}{cc} L_{id} & =E_{x∼X}\left[{∥T_{Y\to X}\left(x,M_{X}^{\prime }\right)-x∥}_{1}\right]\\ \; & \;+E_{y∼Y}\left[{∥T_{X\to Y}\left(y,M_{Y}^{\prime }\right)-y∥}_{1}\right] \end{array}$$

where $\left\{M_{X}^{\prime },M_{Y}^{\prime }\right\}$ represents the attention maps inferred from real images {x,y} using the networks designed for opposite domains.

#### Total Loss

The total loss’ objective is as follows:

(8) $$L_{total}=L_{adv1}+L_{adv2}+\lambda_{cyc}L_{cyc}+\lambda_{id}L_{id}$$

where $\lambda_{cyc}$ and $\lambda_{id}$ are hyperparameters to control the influences of each loss.

## 4. Experiments

In this section, we first compare our method with related methods for day-to-night image translation. Then, we present experiments to investigate the validity of the architecture of the proposed method.

### 4.1. Experimental Environments

#### 4.1.1. Datasets

The proposed method requires daytime and nighttime images, along with the corresponding labels for semantic segmentation and depth during training. However, there is currently no available dataset that encompasses all these requirements. Consequently, we conducted training using data from two distinct datasets: the Berkeley Deep Drive (BDD) dataset [13] and the Cityscapes dataset [14].

The Berkeley Deep Drive dataset comprises 10,000 RGB images capturing diverse driving scenarios (e.g., highway, urban area, bridge, and tunnel). The dataset includes variations in weather conditions and the time of day. Semantic segmentation labels are provided for these images. The image resolutions are 1280×720 pixels.

The Cityscapes dataset offers 5000 RGB daytime images showcasing various driving environments, accompanied by multiple annotations. In particular, the dataset includes disparity information that can be converted to depth. The image resolutions are 2048×1024 pixels.

During the training of the network outlined in Section 3.1, we utilized semantic segmentation labels from the BDD dataset and depth labels from the Cityscapes dataset. The image translation network described in Section 3.2 was trained using both daytime and nighttime images from the BDD dataset.

#### 4.1.2. Implementation Settings

Our networks were implemented based on the architectures of CycleGAN (https://github.com/junyanz/pytorch-CycleGAN-and-pix2pix accessed on 11 January 2024) [22] and CBAM (https://github.com/Jongchan/attention-module accessed on 11 January 2024) [55], using the PyTorch framework [63], and were trained on a single NVIDIA GeForce RTX 3090 GPU. The hyperparameters and training details are as follows.

- Learning rate: fixed at lr=0.0002 for the initial 100 epochs and then linearly decayed to lr=0 over the next 100 epochs.
- Batch size: set to 4.
- Optimization algorithm: Adam with $\beta_{1}=0.5$, $\beta_{2}=0.999$.

During training, we randomly sampled 1000 daytime images with semantic segmentation labels and 1000 nighttime images from the BDD dataset. In addition, 1000 daytime images with depth labels were randomly selected from the Cityscapes dataset. The images from the BDD dataset and the Cityscapes dataset were randomly cropped to 512×512 pixels and 824×824 pixels, respectively. Subsequently, the images were resized to 256×256 pixels after the cropping. For testing, 1000 daytime images were randomly sampled from the BDD dataset, and these images were center-cropped to 512×512 pixels before being resized to 256×256 pixels.

#### 4.1.3. Comparison

We compare the proposed method with the following models.

- CycleGAN [22] introduced cycle consistency loss for unpaired image-to-image translation.
- SemGAN [41] adopted semantic consistency loss to maintain semantic information during image-to-image translation.
- AugGAN [43,44] learned image translation and semantic segmentation simultaneously.
- Lee et al. [64] transfer-learned the weights of semantic segmentation networks to the day-to-night image translation networks.
- UNIT [27] achieves unsupervised image-to-image translation through VAE [16] from different domains that share a latent space.
- MUNIT [30] is a multimodal unsupervised image-to-image translation method that is an extension of UNIT [27].

#### 4.1.4. Evaluation Metrics

We evaluate the proposed and compared methods using the following metrics for quantitative comparisons.

- The Fréchet Inception Distance (FID) [65] measures the Fréchet distance between the distributions of features extracted from the Inception-V3 network [66] for real and generated images.
- The Kernel Inception Distance (KID) [67] uses the calculation of the squared Maximum Mean Discrepancy (MMD) by comparing the Inception-V3 [66] features of the real and translated samples. This comparison is conducted through the application of a polynomial kernel.
- The Learned Perceptual Image Patch Similarity (LPIPS) metric [68], utilized to assess the diversity of an image set, computes the average feature distances between all pairs of images. Specifically, it gauges the translation diversity by evaluating the similarity between distinct deep features extracted from the pre-trained AlexNet [69].

### 4.2. Comparison to the Related Work

We conducted experiments to compare our proposed method with the related works as mentioned in Section 4.1.3.

**Figure 4:**
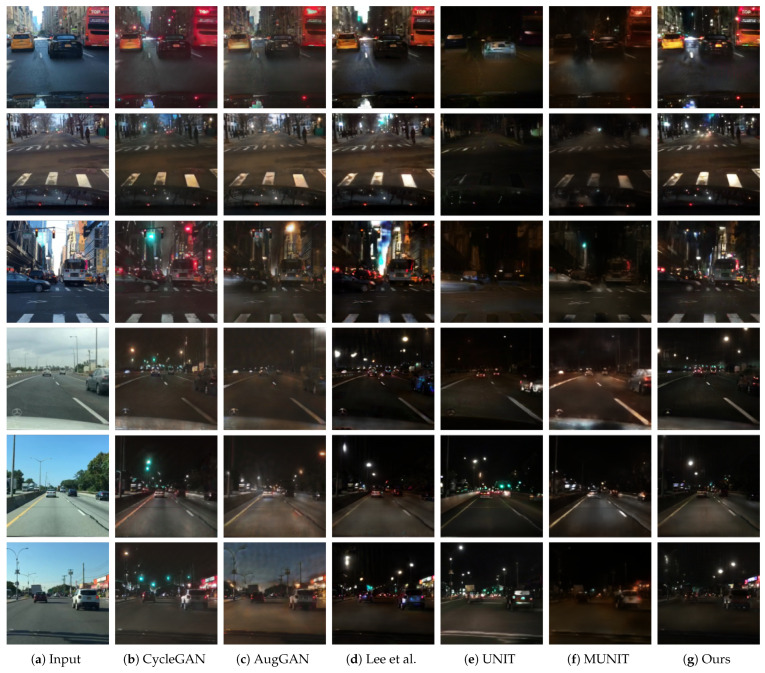
Qualitative comparison with related works. The figure shows, from left to right, the input images in the source domain, followed by the day-to-night image translation results from CycleGAN [22], SemGAN [41], AugGAN [43,44], Lee et al. [64], UNIT [27], MUNIT [30], and our method, respectively.

Figure 4 shows the day-to-night image translation results of each method. CycleGAN [22], Lee et al.’s method [64], UNIT [27], and the proposed method generate expressions of streetlights, while other methods do not generate these expressions. Furthermore, our method generates visually more detailed expressions (i.e., color, shape, and position) of the streetlights than CycleGAN [22], Lee et al.’s method [64] and UNIT [27]. Translation failures in the sky area, sandwiched between buildings, are observed from the results of CycleGAN [22], AugGAN [43,44], and Lee et al.’s method [64]. UNIT [27] and MUNIT [30] appear to fail in their translations, as they darken the entire scene, giving the impression of inverting the colors in the images. In contrast, the proposed model’s results demonstrate the preservation of color information for each object in the input image. The results of our method show the car and road in front of the ego vehicle being illuminated by headlights.

Table 1 shows the quantitative evaluation with FID [65], KID [67], and Diversity (LPIPS [68]). Our method outperforms other methods in both the FID [65] and KID [67] metrics, which evaluate the realism of the results. AugGAN [43,44] obtained the highest score in Diversity (LPIPS [68]). However, it is crucial to note that achieving a high score in Diversity (LPIPS [68]) does not necessarily indicate a suitable result, as this metric does not consider the realism aspect. This point is underscored by the presence of images in the bottom row of AugGAN’s outputs in Figure 4. Here, the translation between daytime and nighttime failed, and the increased Diversity (LPIPS [68]) can be interpreted as a result of images closer to daytime (which lack realism). Therefore, Diversity (LPIPS [68]) metrics should be evaluated comprehensively alongside realism assessments.

In this context, our proposed model received the highest evaluations in both the quantitative and qualitative assessments of realism. Simultaneously, it recorded values close to the Diversity (LPIPS [68]) of real night images. Hence, our proposed model can be deemed to have performed the best overall.

### 4.3. Network Settings

We examined different configurations of the proposed method to optimize the architectural composition. Initially, we analyzed the impact of combinations of attention maps. Subsequently, we demonstrated the effectiveness of sharing feature maps within the decoder during the day-to-night cycle, and alternatively calculating them separately within the night-to-day cycle.

#### 4.3.1. Pipelines of the Semantic and Geometric Feature Maps

**Figure 5:**
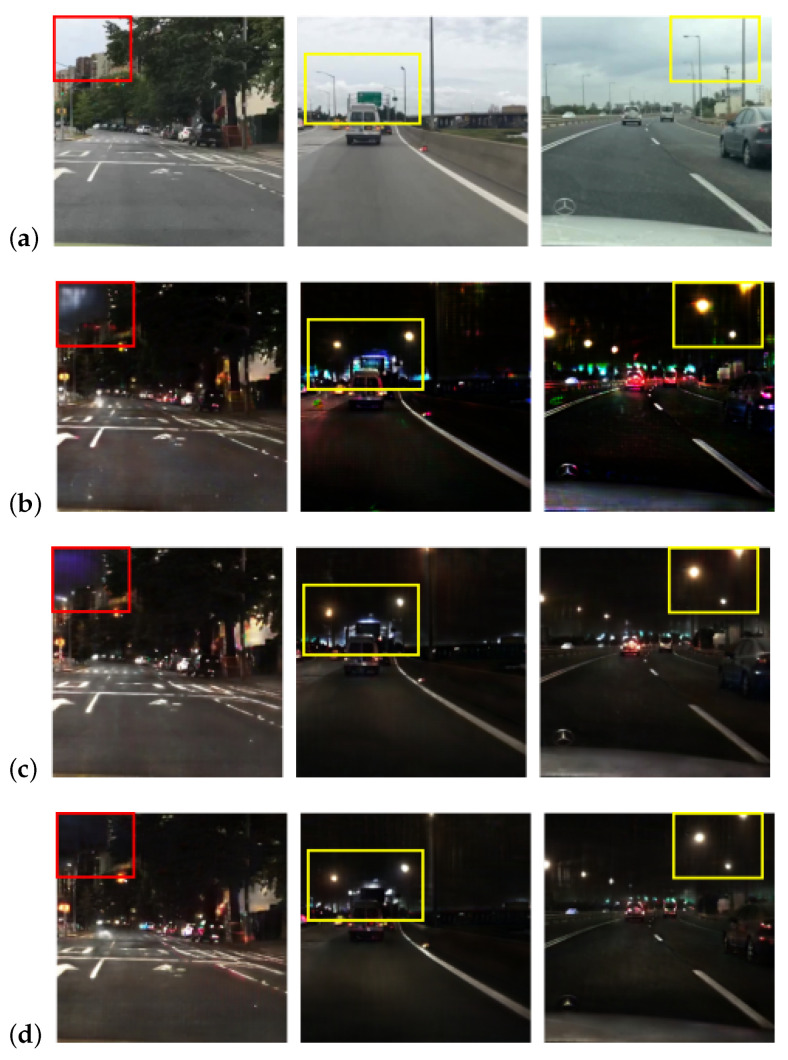
Comparison of pipelines for feature maps from semantic segmentation and depth generators. We present (**a**) input images from the source domain, followed by the outputs from networks employing three distinct pipelines with attention modules. The pipeline in (**b**) shares feature maps during both day-to-night and night-to-day cycles, while that in (**c**) shares feature maps exclusively during the day-to-night cycle, without adopting attention maps during the night-to-day cycle. Additionally, the pipeline in (**d**) shares feature maps only during the day-to-night cycle and separately extracts them during the night-to-day cycle. The red-colored boxes denote the sky area between buildings or trees, while the yellow-colored boxes highlight areas containing street lights.

The proposed method, as shown in Figure 2b, incorporates the sharing of feature maps from the semantic segmentation and depth estimation decoders during the day-to-night cycle. While a similar process could be applied to the night-to-day cycle, estimating semantic segmentation and depth maps from nighttime images poses a challenge. There is a concern that sharing low-accuracy estimates may mislead the reconstruction process. To address this, we conducted experiments with three types of feature map pipelines throughout the night-to-day cycle. Figure 5 illustrates the visual results of these three pipelines.

In the night-to-day cycle, the expressions of the sky and streetlights vary based on the chosen pipeline. Sharing the feature maps or not adopting attention maps within the night-to-day cycle can lead to expression failures in the sky. On the other hand, when we separately extract the feature maps for translation and reconstruction during the night-to-day cycle, more detailed expressions of streetlights are observed. Table 2 indicates that the translated images obtained by separately extracting the feature maps during the night-to-day cycle are more realistic in all metrics.

We adopt the pipeline that shares the feature maps on the day-to-night cycle and extracts them separately on the night-to-day cycle for our proposed method.

#### 4.3.2. Attention Modules

The proposed method aimed to enhance the semantic and geometric information for the day-to-night image translation network. As a means of enhancing the semantic and geometric information, we introduced attention maps derived from relevant information in the input images, which were then utilized in the image translation network.

We assumed that the semantic and geometric information was able to be extracted as feature maps by the decoders trained to infer the semantic segmentation and depth. Based on this assumption, we generated attention maps from the feature maps calculated by each decoder. In our method, one image translation network adopts two different sizes of attention maps for semantic and geometric information, respectively: ${\left\{m_{T}^{k}\right\}}_{k\in \{Seg_{1},Dep_{1},Seg_{2},Dep_{2}\}}$, where T∈{X,Y} indicates the time domain. Each attention map is inferred from a different size or type of feature map. $\left\{m_{T}^{Seg_{1}},m_{T}^{Seg_{2}}\right\}$ and $\left\{m_{T}^{Dep_{1}},m_{T}^{Dep_{2}}\right\}$ are generated from the feature maps of the semantic segmentation and depth decoders. Moreover, $\left\{m_{T}^{Seg_{1}},m_{T}^{Dep_{1}}\right\}$ are generated from relatively small-sized feature maps, whereas $\left\{m_{T}^{Seg_{2}},m_{T}^{Dep_{2}}\right\}$ are inferred from relatively large-sized feature maps.

**Figure 6:**
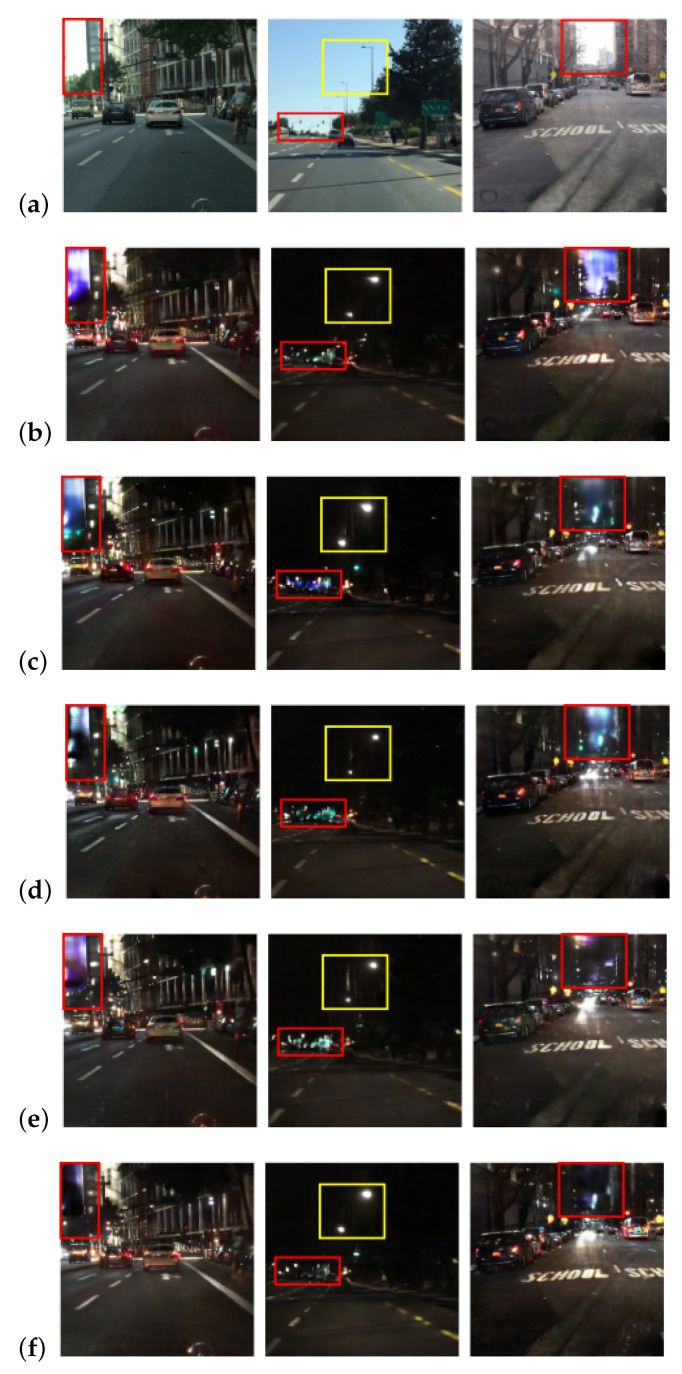
Comparison for combinations of attention maps. Each row shows, from top to bottom, (**a**) input images from the source domain and subsequently the outputs from networks employing four distinct combinations of attention maps. The combinations are (**b**) small-sized attention maps $\left\{m^{Seg_{1}},m^{Dep_{1}}\right\}$, (**c**) large-sized attention maps $\left\{m^{Seg_{2}},m^{Dep_{2}}\right\}$, (**d**) generated from feature maps of the depth decoder $\left\{m_{T}^{Dep_{1}},m_{T}^{Dep_{2}}\right\}$, (**e**) generated from feature maps of the semantic segmentation decoder $\left\{m_{T}^{Seg_{1}},m_{T}^{Seg_{2}}\right\}$, and (**f**) all feature maps $\left\{m^{Seg_{1}},m^{Dep_{1}},m^{Seg_{2}},m^{Dep_{2}}\right\}$. The red-colored boxes denote the sky area between buildings or trees, while the yellow-colored boxes highlight areas containing streetlights.

We conducted an experiment to verify the effects of combinations of these attention maps. Several combinations of different types and sizes of attention maps were applied to the image-to-image translation networks. Figure 6 and Table 3 present visual and quantitative comparisons of the results for each network combination.

In the visual comparison, translation failures in the sky area, sandwiched between the buildings, are observed in all other combinations except the proposed method. Additionally, the expressed size of the streetlight depends on the combination of attention maps, and the streetlights tended to appear larger when the image translation network adopted combinations including $\left\{m_{T}^{Seg_{2}},m_{T}^{Dep2}\right\}$.

The quantitative results indicate that the image translation network with all types and sizes of attention maps achieved the best results in both metrics.

With both the visual and quantitative results, we utilize all attention maps for our proposed method.

### 4.4. Discussion

Based on our assumption that the semantic segmentation and depth decoders can extract the semantic and geometric information from the input image, our method infers the semantic and geometric attention maps from the related feature maps extracted by the decoders. The attention maps were created from two different sizes of feature maps calculated from each decoder and applied to the image translation network. To investigate the necessity of these diverse types and sizes of attention maps for image translation, we conducted an experiment. The experimental results show that employing all types of attention maps yielded the best results in both the qualitative and quantitative evaluations.

Additionally, an experiment was performed to identify the optimal conditions for the pipeline of decoder feature maps in the image translation and reconstruction networks during the night-to-day cycle. The comprehensive results validate the effectiveness of our approach, demonstrating that introducing attention maps inferred from two different-sized feature maps from each decoder and extracting feature maps separately for the image translation and reconstruction networks during the night-to-day cycle yield the best performance.

Comparative evaluations against CycleGAN [22], SemGAN [41], AugGAN [43,44], Lee et al.’s method [64], UNIT [27], and MUNIT [30] substantiate the notion that our proposed model outperforms these methods in both quantitative and qualitative assessments.

Looking ahead, future efforts should include exploring the application of the proposed model to adverse weather conditions, such as rain or fog. In addition, research efforts are necessary that focus on leveraging translated images for the enhancement or evaluation of model training.

## 5. Conclusions

In this paper, we present an unpaired image-to-image translation network with semantic and geometric attention maps. We propose to first pre-train the multi-task network for the estimation of semantic segmentation and depth maps and then utilize the extracted feature maps from these pre-trained decoders to infer semantic and geometric attention maps. We apply these attention maps to the image-to-image translation network. The experiments indicate both qualitative and quantitative performance gains with our proposed method. 

## Figures and Tables

**Table 1:** Quantitative evaluation with FID [65], KID [67], and Diversity (LPIPS [68]).

Method	FID ↓	KID$\times_{100}↓$	Diversity ↑
CycleGAN [22]	35.528	1.321	0.597
SemGAN [41]	35.256	1.309	0.605
AugGAN [43,44]	57.723	4.013	**0.631**
Lee et al. [64]	39.598	1.727	0.587
UNIT [27]	32.661	1.080	0.586
MUNIT [30]	69.968	5.791	0.578
Ours	**31.245**	**0.898**	0.586
Real night images	19.673	0.022	0.601

The up and down arrows next to the metrics indicate that a larger and smaller numerical value corresponds to a better outcome, respectively. The bold numbers highlight the best results.

**Table 2:** Quantitative evaluation of pipelines of the feature maps for attention maps.

Pipeline	Feature Maps for Attention Maps		FID ↓	KID$\times_{100}↓$	Diversity ↑
	Day-to-Night Cyc	Night-to-Day Cyc			
1	share	share	35.963	1.293	**0.595**
2	share	no attention	34.856	1.216	**0.596**
3	share	separate	**31.245**	**0.898**	0.586

The up and down arrows next to the metrics indicate that a larger and smaller numerical value corresponds to a better outcome, respectively. The bold numbers highlight the best results.

**Table 3:** Quantitative evaluation of attention map combinations.

Method	Adopted Attention Maps ^1^				FID ↓	KID$\times_{100}↓$	Diversity ↑
	$m_{T}^{{\mathrm{Seg}}_{1}}$	$m_{T}^{{\mathrm{Dep}}_{1}}$	$m_{T}^{{\mathrm{Seg}}_{2}}$	$m_{T}^{{\mathrm{Dep}}_{2}}$			
Proposed method w/o $m_{T}^{Seg_{2}},m_{T}^{Dep_{2}}$ ^2^	✓	✓	-	-	33.696	1.118	**0.591**
Proposed method w/o $m_{T}^{Seg_{1}},m_{T}^{Dep_{1}}$ ^3^	-	-	✓	✓	33.786	1.161	0.589
Proposed method w/o $m_{T}^{Dep_{1}},m_{T}^{Dep_{2}}$ ^4^	✓	-	✓	-	33.365	1.132	**0.591**
Proposed method w/o $m_{T}^{Seg_{1}},m_{T}^{Seg_{2}}$ ^5^	-	✓	-	✓	35.105	1.324	0.590
Proposed method	✓	✓	✓	✓	**31.245**	**0.898**	0.586

^1^ *T* indicates time domain: T∈{X,Y}. ^2^ The network only adopts small-sized attention maps. ^3^ The network only adopts large-sized attention maps. ^4^ The network only adopts semantic attention maps. ^5^ The network only adopts geometric attention maps. The up and down arrows next to the metrics indicate that a larger and smaller numerical value corresponds to a better outcome, respectively. The bold numbers highlight the best results.

## Data Availability

The data presented in this study are available on request from the corresponding authors. Please note that the data are not publicly accessible due to contractual obligations associated with the project. These data were derived from the following resources available in the public domain: BDD dataset (https://www.vis.xyz/bdd100k/ accessed on 11 January 2024), Cityscapes dataset (https://www.cityscapes-dataset.com/ accessed on 11 January 2024).
